# When the Transition Goes Wrong: A Rare Case of Diabetic Ketoalkalosis After Transitioning to Tirzepatide in Insulin-dependent Type 2 Diabetes

**DOI:** 10.7759/cureus.87031

**Published:** 2025-06-30

**Authors:** Waleed Sultan, Jeanne Spencer

**Affiliations:** 1 Family Medicine, Conemaugh Memorial Medical Center, Johnstown, USA

**Keywords:** antihyperglycemic agents, diabetic ketoacidosis, diabetic ketoal glp-1/gip receptor agonist, diabetic ketoalkalosis, incretin-based therapy, insulin discontinuation, metabolic alkalosis, tirzepatide, type 2 diabetes mellitus

## Abstract

Type 2 diabetes mellitus (T2DM) often requires pharmacological management, with newer agents like glucagon-like peptide-1 (GLP-1) receptor agonists (GLP-1 RA) and dual gastric inhibitory polypeptide (GIP)/GLP-1 receptor agonists offering metabolic and cardiovascular benefits. However, transitioning from insulin therapy to these agents can lead to rare complications, such as diabetic ketoalkalosis, a metabolic disturbance marked by simultaneous ketosis and alkalosis.

We report an unusual case of diabetic ketoalkalosis, a mixed acid-base disorder, in a 57-year-old female patient with insulin-dependent T2DM following a switch to Mounjaro (tirzepatide). The patient presented with nausea, vomiting, elevated anion gap, ketosis, and a slightly alkalotic venous pH. This was attributed to insulin deficiency and vomiting-induced acid loss and bicarbonate retention. Management with fluids, electrolytes, and insulin led to rapid recovery. This case highlights the need for gradual insulin tapering and close monitoring while transitioning from insulin therapy to GIP/GLP-1 receptor agonists.

## Introduction

Type 2 diabetes mellitus (T2DM) is a chronic metabolic disease marked by insulin resistance and progressive β-cell dysfunction. Pharmacologic therapy is often required to maintain glycemic control, with insulin traditionally serving as a cornerstone in patients with significant hyperglycemia or β-cell failure [1]. In recent years, incretin-based therapies, including glucagon-like peptide-1 receptor agonists (GLP-1 RA) and dual glucose-dependent insulinotropic polypeptide/glucagon-like peptide-1 receptor agonists (GIP/GLP-1 RA), have gained traction due to their benefits on weight loss, cardiovascular risk, and renal protection [2-4].

Despite these advantages, transitioning insulin-dependent patients to these newer agents may pose metabolic risks. One rare but increasingly recognized complication is diabetic ketoalkalosis, a mixed acid-base disorder characterized by concurrent ketoacidosis and metabolic alkalosis [5,6]. This case report presents a patient with T2DM who developed diabetic ketoalkalosis following abrupt discontinuation of basal insulin during the initiation of tirzepatide (Mounjaro), emphasizing the need for gradual insulin tapering and close monitoring while transitioning from insulin to GIP/GLP-1 RA in high-risk individuals.

## Case presentation

A 57-year-old female patient with a history of insulin-dependent type 2 diabetes mellitus presented to the emergency department with intractable nausea and vomiting. She reported that these symptoms had been ongoing but had significantly worsened over the preceding several weeks. This exacerbation followed a recent change in her diabetes regimen; She had been transitioned from a combination of long-acting insulin (40 units nightly) and short-acting insulin (10 units before meals) to tirzepatide (Mounjaro), a dual GLP-1/GIP receptor agonist, approximately seven weeks prior to presentation. The transition was made due to persistently uncontrolled blood glucose levels, with a recent hemoglobin A1c of 12%. The patient completely discontinued the insulin once she started the tirzepatide and hasn't been to a follow-up visit, with poor home glucose monitoring since the transition.

On physical examination, the patient appeared dehydrated, exhibited deep and rapid respirations (Kussmaul breathing), and had a fruity odor on her breath. Initial laboratory evaluation revealed significant metabolic derangements, including findings consistent with both ketoacidosis and metabolic alkalosis, as detailed in Table 1.

**Table 1:** Initial labs in the emergency department showed a mixed metabolic state.

Lab Test	Result	Reference Range	Interpretation
Random blood glucose	320 mg/dL	70–140 mg/dL	Elevated (Hyperglycemia)
Anion gap	23 mmol/L	8–16 mmol/L	Elevated (metabolic acidosis)
Beta-hydroxybutyrate	6.7 mmol/L	<0.6 mmol/L	Significantly elevated (ketosis)
Lipase	30 U/L	12-52 U/L	Normal
Serum potassium	3.2 mmol/L	3.5–5.0 mmol/L	Low (mild hypokalemia)
Serum osmolality	340 mOsm/kg	275–295 mOsm/kg	Elevated (hyperosmolar state)
Venous pH	7.45	7.32–7.42	Slightly alkalotic (suggests metabolic alkalosis)

The elevated anion gap and markedly raised beta-hydroxybutyrate were consistent with diabetic ketosis with coexisting metabolic alkalosis (mixed metabolic state). Metabolic alkalosis was likely secondary to prolonged vomiting, which masked the typical acidosis seen in diabetic ketoacidosis (DKA).

The patient was managed with a two-liter IV normal saline bolus; she was restarted on her long-acting insulin at a reduced dose of 25 units and a low-dose sliding scale. The patient responded well to this conservative approach and demonstrated rapid clinical improvement and normalization of anion gap and venous pH within 18 hours.

To further evaluate the underlying etiology and rule out type 1 diabetes, her C-peptide level was consistent with type 2 diabetes. Since the patient's condition stabilized quickly and she remained clinically well, she was discharged home on her prior long-acting insulin and sliding scale insulin three times daily, with plans for close outpatient follow-up and monitoring.

## Discussion

**Figure 1:**
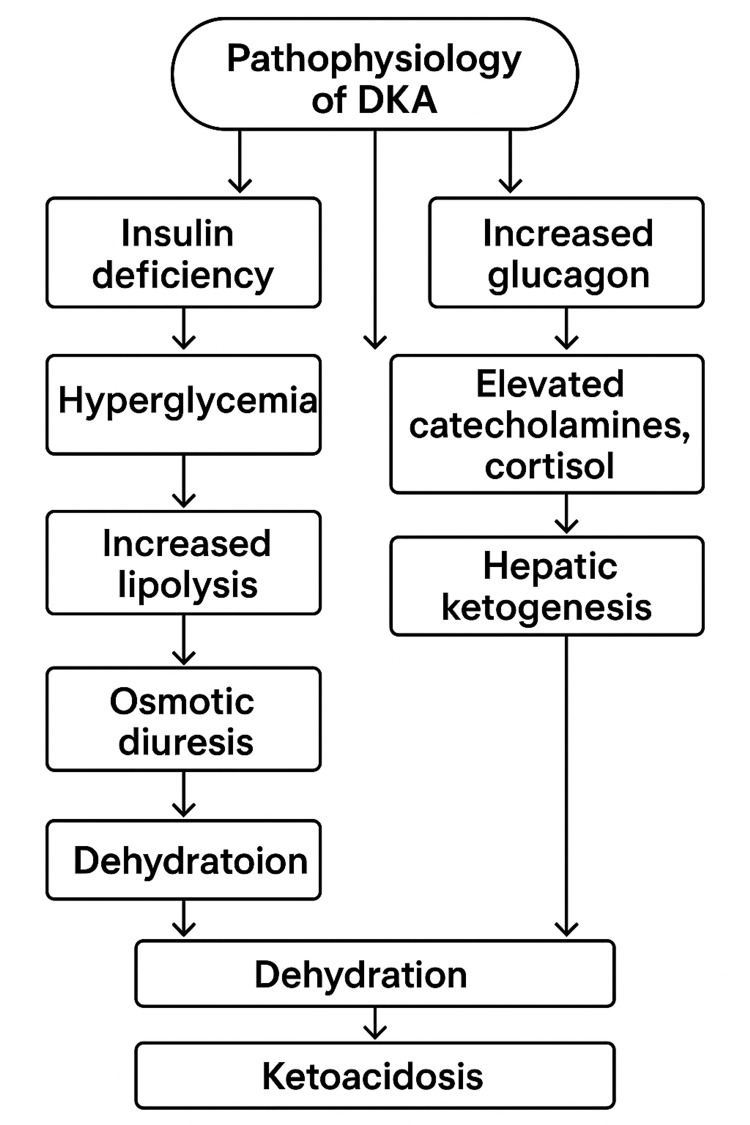
Pathophysiology of DKA. DKA: Diabetic ketoacidosis Image credit: Author-created.

DKA is a potentially life-threatening condition that primarily affects individuals with type 1 diabetes but can also occur in type 2 diabetes. It results from severe insulin deficiency and increased levels of counter-regulatory hormones, leading to hyperglycemia, ketosis, and metabolic acidosis (Figure 1) [1].

Glucagon-like peptide-1 (GLP-1) and glucose-dependent insulinotropic polypeptide (GIP) receptor agonists (RAs) are a class of drugs used in type 2 diabetes management. They mimic the effects of incretin hormones (GLP-1 and GIP), which are naturally released from the gut in response to food intake, enhancing glucose regulation [2,3]. The mechanism of action is summarized in Table 2.

**Table 2:** Mechanisms of Action of GLP-1 and GIP Receptor Agonists. GLP-1 RA: Glucagon-like peptide-1 receptor agonist; GIP RA:Glucose-dependent insulinotropic polypeptide receptor agonist; CNS: central nervous system; CV: cardiovascular

Mechanism	GLP-1 Receptor Agonist	GIP Receptor Agonist
Primary Target	GLP-1 receptors (β-cells, CNS, stomach, heart)	GIP receptors (β-cells, adipose tissue, CNS)
Insulin Secretion	↑ Glucose-dependent insulin release	↑ Glucose-dependent insulin release
Glucagon Secretion	↓ Suppressed postprandial glucagon	Context-dependent (↑ during hypoglycemia, ↓ postprandially)
Gastric Emptying	↓ Delayed	Minimal effect
Appetite / Satiety	↑ Satiety, ↓ food intake	Modulates appetite; effect amplified when combined with GLP-1 RA
Body Weight	↓ Weight (via appetite suppression)	Neutral or ↑ when alone; ↓ when combined with GLP-1 RA
Adipose Tissue Effects	↓ Lipogenesis	↑ Lipid buffering, ↑ insulin sensitivity, ↑ storage capacity
β-cell Function	↑ Proliferation & survival	↑ Function (less robust alone)
Cardiometabolic Effects	↓ CV events, ↓ inflammation	Under investigation

Switching from insulin to GLP-1/GIP receptor agonists (e.g., tirzepatide) may benefit patients with type 2 diabetes, especially those needing weight loss, better glycemic control, or reduced hypoglycemia. The transition should be gradual and individualized. It is suitable for patients on basal insulin with poor control or frequent hypoglycemia, but is not recommended for those with type 1 diabetes or severe insulin deficiency [3]. The SURPASS-5 (Study of Tirzepatide in Participants With Type 2 Diabetes on Insulin Glargine) trial demonstrated that switching to tirzepatide from insulin therapy significantly reduced insulin requirements [7]. Based on these findings, it is recommended to gradually reduce insulin doses when initiating tirzepatide to prevent hypoglycemia and optimize glycemic control [7]. For patients with an HbA1c of 8% or less, a 20% reduction in insulin dose upon starting tirzepatide may be appropriate. While exact dose adjustments should be individualized, monitoring blood glucose levels and making gradual insulin titration is essential for achieving the best outcomes [3,7].

While transitioning from insulin to GLP-1/GIP receptor agonists (e.g., tirzepatide, semaglutide, dulaglutide) offers benefits such as improved glycemic control, weight loss, and reduced hypoglycemia risk, some complications can arise during the switch, as shown in Table 3.

**Table 3:** Possible complications on switching from insulin to GLP1/GIP-RAs and prevention methods. GLP-1-RA: Glucagon-like peptide-1 receptor agonist; GIP-RA: glucose-dependent insulinotropic polypeptide receptor agonist; DKA: diabetic ketoacidosis

Complication	Cause	Prevention Strategy
Hyperglycemia	Rapid insulin reduction or discontinuation	Gradual insulin tapering; frequent blood glucose monitoring
Ketosis / Diabetic Ketoacidosis (DKA)	Excessive insulin reduction in insulin-dependent patients	Avoid abrupt basal insulin discontinuation; monitor ketones
Gastrointestinal Side Effects	GLP-1-induced delayed gastric emptying	Start at a low dose, then titrate slowly
Hypoglycemia	Insulin dose is not reduced appropriately when starting GLP-1/GIP RA	Reduce insulin dose appropriately at initiation; monitor glucose levels
Dehydration and Electrolyte Imbalance	Vomiting or reduced food intake	Encourage hydration; monitor electrolytes
Weight Loss-Related Muscle Loss	Reduced appetite, rapid weight loss, decreased protein intake	Ensure adequate protein intake; encourage resistance exercise
Pancreatitis	GLP-1 receptor activation in susceptible patients	Monitor for severe abdominal pain; discontinue if pancreatitis is suspected.

Diabetic ketoalkalosis (DKA-Alk) is a rare condition where metabolic alkalosis coexists with DKA, creating a mixed acid-base disturbance. While DKA is driven by anion gap metabolic acidosis due to ketone buildup from insulin deficiency, vomiting can cause gastric acid loss, leading to bicarbonate retention and hypochloremic alkalosis. Other contributors include osmotic diuresis, respiratory compensation, and excessive bicarbonate use. Patients may show symptoms of both conditions, such as polyuria, nausea, Kussmaul breathing, and confusion. Treatment includes fluid resuscitation, electrolyte replacement, and cautious insulin therapy; bicarbonate is reserved for pH<6.9 [5,6].

**Figure 2:**
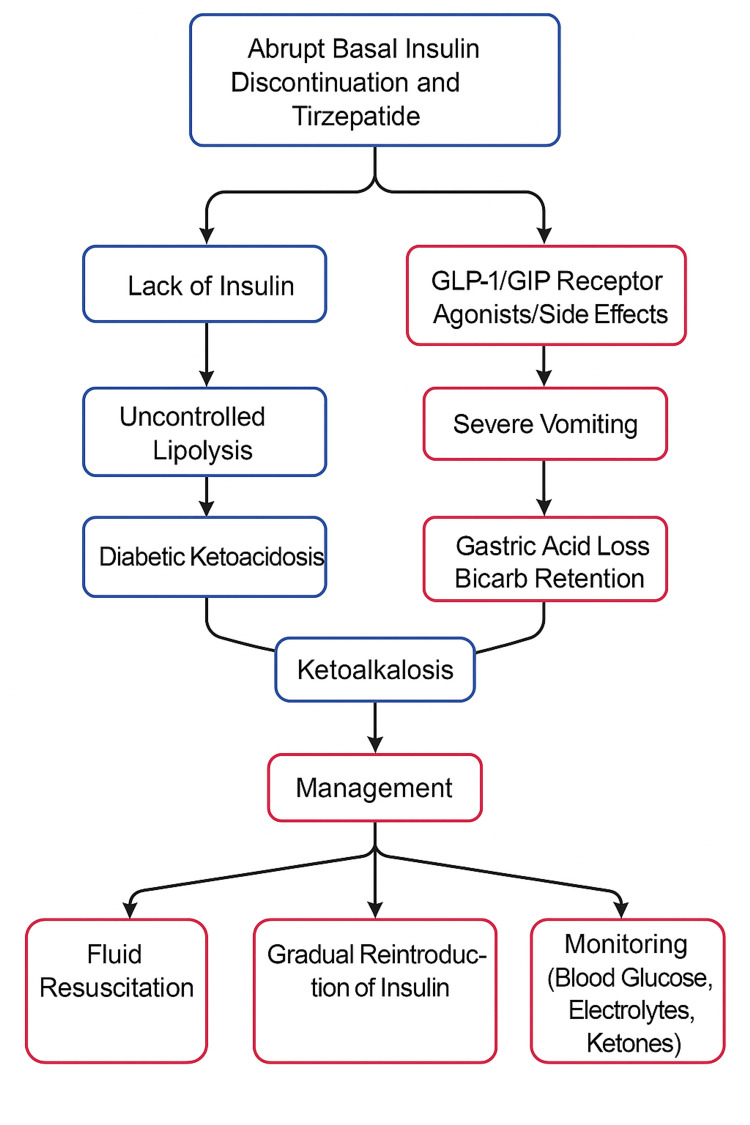
Pathophysiology of mixed metabolic state, diabetic ketolakalosis, and proper management. Image credit: Author-created.

In our case, abrupt basal insulin discontinuation during the transition to GLP-1/GIP receptor agonists triggered DKA-Alk. Insulin deficiency led to ketosis, while vomiting caused alkalosis. The patient improved with fluids, insulin, and electrolyte monitoring. The pathophysiology and management are illustrated in Figure 2.

## Conclusions

Diabetic ketoalkalosis is a rare but serious complication that can occur during transitions from insulin to GLP-1/GIP receptor agonists. In this case, the abrupt discontinuation of insulin and GLP-1/GIP RA-induced vomiting triggered a mixed acid-base disorder. The patient improved with careful management, including fluid resuscitation, insulin reintroduction, and electrolyte monitoring. This case underscores the importance of gradual insulin tapering and close monitoring to prevent metabolic complications during such transitions. Given the limited literature on diabetic ketoalkalosis during incretin-based transitions, further studies and reports are needed to better understand its incidence, pathophysiology, and optimal management strategies.
